# A Docosahexaenoic Acid Derivative (*N*-Benzyl Docosahexaenamide) as a Potential Therapeutic Candidate for Treatment of Ovarian Injury in the Mouse Model

**DOI:** 10.3390/molecules27092754

**Published:** 2022-04-25

**Authors:** Lirong Guo, Qing Gao, Jieqiong Zhu, Xiaobao Jin, Hui Yin, Tao Liu

**Affiliations:** 1 Guangdong Provincial Key Laboratory of Pharmaceutical Bioactive Substances, School of Biosciences and Biopharmaceutics, Guangdong Pharmaceutical University, Guangzhou 510006, China; glr19951215@163.com (L.G.); qinggao0403@foxmail.com (Q.G.); zhujieqiong999@foxmail.com (J.Z.); jinxf2001@163.com (X.J.); 2 School of Pharmacy, Guangdong Pharmaceutical University, Guangzhou 510006, China

**Keywords:** docosahexaenoic acids, ovary, granulosa cells, cyclophosphamide, macamide

## Abstract

Commonly used clinical chemotherapy drugs, such as cyclophosphamide (CTX), may cause injury to the ovaries. Hormone therapies can reduce the ovarian injury risk; however, they do not achieve the desired effect and have obvious side effects. Therefore, it is necessary to find a potential therapeutic candidate for ovarian injury after chemotherapy. *N*-Benzyl docosahexaenamide (NB-DHA) is a docosahexaenoic acid derivative. It was recently identified as the specific macamide with a high degree of unsaturation in maca (*Lepidium meyenii*). In this study, the purified NB-DHA was administered intragastrically to the mice with CTX-induced ovarian injury at three dose levels. Blood and tissue samples were collected to assess the regulation of NB-DHA on ovarian function. The results indicated that NB-DHA was effective in improving the disorder of estrous cycle, and the CTX+NB-H group can be recovered to normal levels. NB-DHA also significantly increased the number of primordial follicles, especially in the CTX+NB-M and CTX+NB-H groups. Follicle-stimulating hormone and luteinizing hormone levels in all treatment groups and estradiol levels in the CTX+NB-H group returned to normal. mRNA expression of ovarian development-related genes was positive regulated. The proportion of granulosa cell apoptosis decreased significantly, especially in the CTX+NB-H group. The expression of anti-Müllerian hormone and follicle-stimulating hormone receptor significantly increased in ovarian tissues after NB-DHA treatment. NB-DHA may be a promising agent for treating ovarian injury.

## 1. Introduction

Docosahexaenoic acid (DHA) is mainly used in the form of DHA-triglycerides or DHA-ethyl esters (DHA-EE). DHA and its derivatives have attracted considerable attention in various research fields, including those involving cell signaling, photoreceptors, the nervous system, and brain development, and exhibit positive effects on ovarian functions and diseases [1]. Additionally, DHA can migrate from different tissues to the ovary during gonadal development and promote the development of model animal ovaries [2]. The DHA content in the lungs is significantly reduced after removal of the ovaries [3]. The dysregulation of ovarian gene expression induced by a high-fat diet is restored by chronic polyunsaturated fatty acid (PUFA)/DHA supplementation [4]. Considering this progress in DHA research for the prevention and mitigation of ovarian-related diseases and functions, the continued discovery and development of new DHA derivatives for ovarian injury remains imperative.

*N*-benzyl docosahexaenamide (NB-DHA) is characterized by the presence of benzylamine-conjugated DHA via an amide bond. Benzylated fatty acids are active ingredients exclusive to maca (*Lepidium meyenii*), named macamides. Notably, NB-DHA had the highest degree of unsaturation among all the identified macamides. Benzylamide is a chemical used to enhance drug activity. For example, *N*-benzyl salinomycin has been reported to exhibit anticancer and antibacterial activities [5]. Deoxynojirimycin derivatives have been studied and can be used as α-glucosidase inhibitors to improve type II diabetes; compound 18, containing an *N*-benzyl amide residue, showed the highest activity [6]. A molecule containing an *N*-benzyl amide residue was screened from a library of compounds, and the results revealed that the synthesized compound is a severe acute respiratory syndrome coronavirus 2 (SARS-CoV-2) replication inhibitor at non-toxic concentrations in vitro and a dual-acting SARS-CoV-2 protease inhibitor against the main protease [7]. Macamides possess various bioactivities, including reproductive health improvement, antioxidation, neuroprotection, anticancer, immunomodulation, and digestive system function-improving activities [8,9,10,11,12,13]. As a newly identified PUFA, NB-DHA can protect the intestinal epithelial barrier and effectively relieve the symptoms of acute colitis in mice [14], while *N*-benzyl eicosapentaenoamide (NB-EPA) alleviates neurobehavioral disorders in neonatal mice with hypoxic-ischemic brain injury through the p53–PUMA signaling pathway [15]. 

Chemotherapeutic drugs, such as clinically available cyclophosphamide (CTX), are toxic to dividing and proliferating cells [16,17]. Chemotherapy with CTX can cause ovarian injury, permanent amenorrhea, and increase the risk of premature menopause [18,19]. The maintenance of ovarian reserve function and prevention of infertility have always been considered by physicians as important prognostic factors during chemotherapy [20,21,22]. Granulosa cells (GCs), as the largest cell group in follicles, play a crucial role in follicle growth and ovarian function regulation. GCs also regulate the development of follicles and are the main functional cells that secrete reproductive hormones [23]. Therefore, it is necessary to find potential therapeutic candidates to relieve and treat ovarian injury, protect GCs, and maintain the growth and development of follicles and ovaries. Although hormone-based treatments for ovarian injury have been adopted in current clinical practice, they do not achieve the desired therapeutic effect, and they also have obvious side effects on the human body [24]. 

In the present study, NB-DHA were synthesized, purified, and administered intragastrically into mice with CTX-induced ovarian injury to assess the regulation of ovarian function. Frequency of occurrence, the stages of the estrous cycle, follicle numbers after H&E staining, four typical sex hormone levels, mRNA expression of five ovarian development-related genes (FOXL2, GDF9, LIF, OCT4, and SCF), granular cell apoptosis ratios, anti-Müllerian hormone (AMH), and follicle-stimulating hormone receptor (FSHR) expression were obtained and analyzed. Terminal deoxynucleotidyl transferase dUTP nick end labeling (TUNEL) fluorescence staining was performed to explore the ovarian injury repair mechanism. 

## 2. Results

### 2.1. Preparation of DHA-EE and NB-DHA

**Figure 1:**
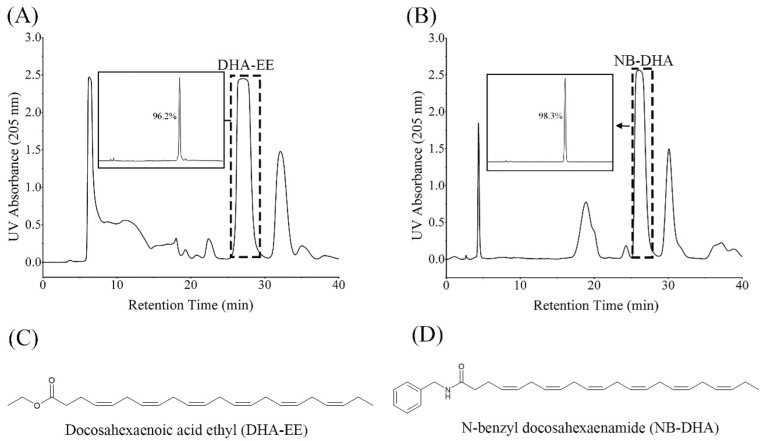
Chromatograms and structural formulas of DHA-EE and NB-DHA. (**A**) Chromatograms of synthetic DHA-EE material and purified DHA-EE sample collected using a semipreparative HPLC system (26.5–30.5 min). (**B**) Chromatograms of synthetic NB-DHA material and purified NB-DHA sample collected using a semipreparative HPLC system (26.0–29.0 min). Structural formulas of DHA-EE (**C**) and NB-DHA (**D**).

Fish oil was hydrolyzed by lipase into free fatty acids, which were used for the synthesis of DHA-EE via the esterification reaction and NB-DHA via the carbodiimide condensation method. The DHA-EE and NB-DHA fractions were collected separately from an HPLC system with elution times of 26.5–30.5 and 26.0–29.0 min, respectively. Both collected fractions were rotary evaporated to dryness and identified by infrared spectroscopy and mass spectrometry (data not shown). Their purities, analyzed by HPLC, were 96.2% (DHA-EE, Figure 1A) and 98.3% (NB-DHA, Figure 1B). The dried samples were used for subsequent animal experiments.

### 2.2. Body/Ovarian Weight and Estrous Cycle Analysis

**Figure 2:**
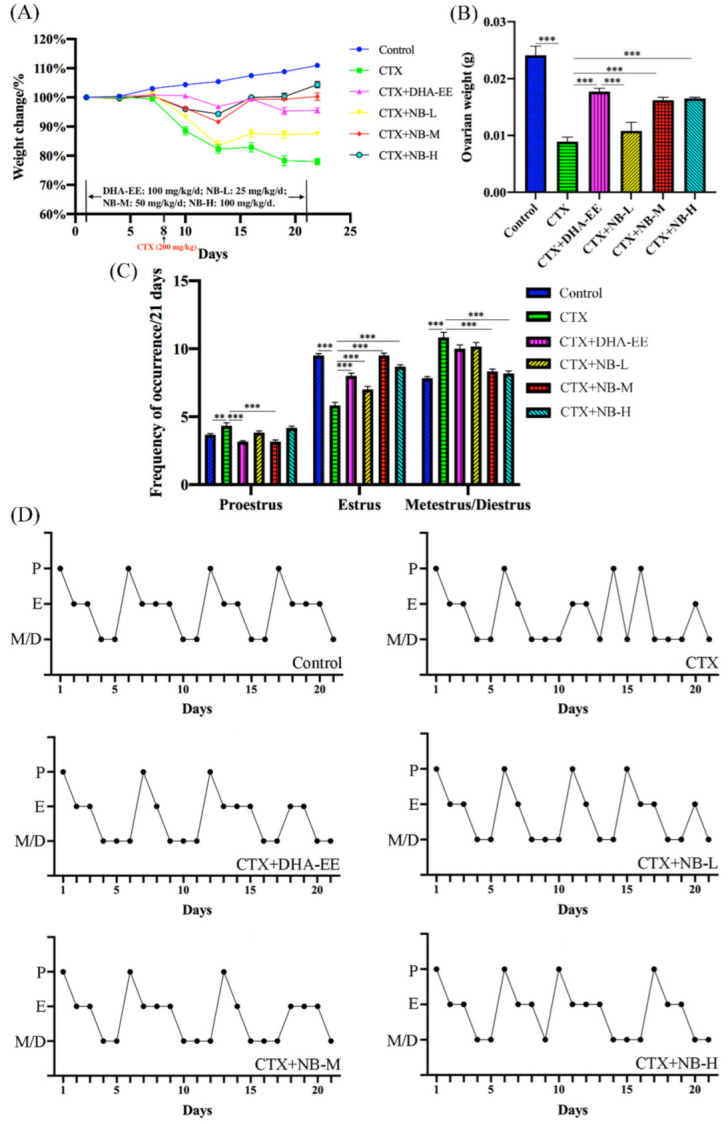
Body weight, ovarian weight, frequency of occurrence, and estrous cycle. Weight Change/% (**A**) and ovarian weight (**B**) measured on days 1, 4, 7, 10, 13, 16, 19, and 22 after administration. Initial average weight of mice was set as 100%. Effect of DHA-EE and NB-DHA on estrous cycles. Frequency of occurrence of cycle stages during the 21 days (n = 6) (**C**) and estrous cycle regularity (**D**). Values in all figures are expressed as the mean ± SEM (x ± sem, n = 6), ** *p* < 0.01, *** *p* < 0.005 (the same below).

As shown in Figure 2A, CTX caused a significant decrease in body weight compared with the control group. The body weight of mice in all DHA-treated groups was significantly higher than that of mice in the CTX group at the end of the 21-day experiment, and body weight recovery was ranked as follows: CTX+NB-H > CTX+NB-M > CTX+DHA-EE > CTX+NB-L. Additionally, compared with the control group, CTX significantly decreased the ovarian weight in the CTX group. After modeling with CTX, ovarian atrophy and ovarian weight decreased significantly. The ovarian weight of mice in the CTX+NB-H, CTX+NB-M, and CTX+DHA-EE groups was higher than that in the CTX group (Figure 2B). CTX is an inducer for the ovarian model that involves prolonging or stagnating the female estrous cycle. The statistical results showed that the time of estrus was shortened, and proestrus, metestrus, and diestrus were prolonged in the CTX group, indicating a disordered estrous cycle in CTX-treated mice (Figure 2C). The 21-day complete estrous cycle of mice was plotted, and the results showed that the average estrous cycle of the control group was 5–6 days. In the CTX group, a complete cycle could not be observed after modeling, but a complete estrous cycle could be observed in each DHA group. DHA-EE and NB-DHA were both effective in alleviating the disorder of the estrous cycle and were ranked as follows: CTX+NB-H > CTX+NB-M > CTX+DHA-EE (Figure 2D).

### 2.3. Follicle Counting and Morphological Analysis

**Figure 3:**
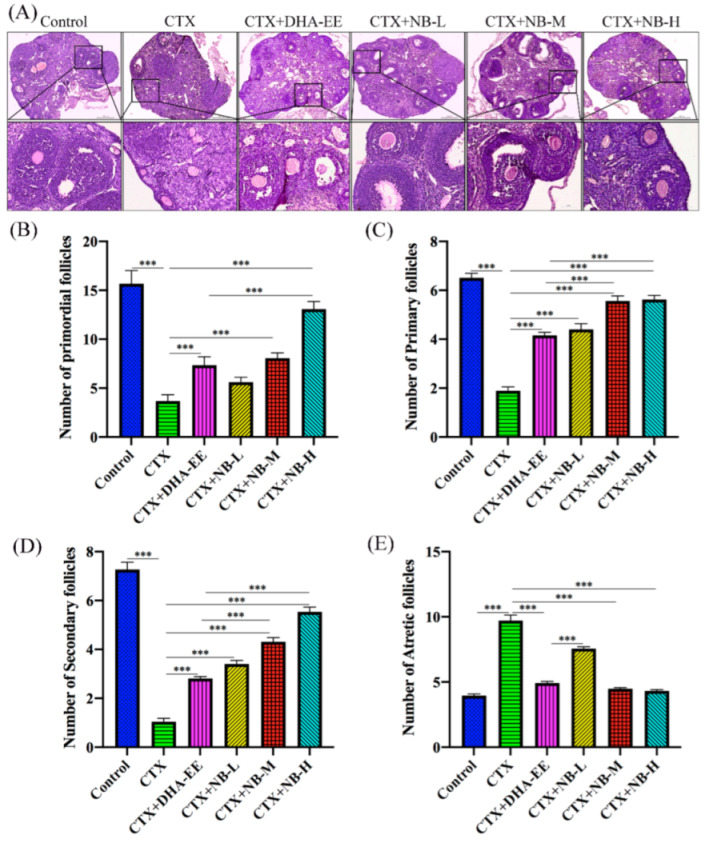
Effect of DHA-EE and NB-DHA on the development of follicles. Follicles after H&E staining (**A**). Magnification 100× and 400×. Scale bar: 250 and 50 μm. Number of different follicles: primordial follicles (**B**), primary follicles (**C**), secondary follicles (**D**), and atretic follicles (**E**). *** *p* < 0.005.

The effects of DHA-EE and NB-DHA on follicular development were analyzed by H&E staining of the ovarian sections (Figure 3A). Abundant healthy follicles were observed in the control group, including primordial, primary, secondary, and atretic follicles, while the distribution of the four types of follicles in the other groups was altered (Figure 3B–E). For example, there were fewer primordial, primary, and secondary follicles, but more atretic follicles in the CTX group than in the control group. Compared with the CTX group, both CTX+DHA-EE and CTX+NB-DHA significantly increased the number of primordial follicles, especially in the CTX+NB-M and CTX+NB-H groups. Moreover, there were fewer atretic follicles in all DHA-treated groups than in the CTX group. The groups ranked as follows: CTX+NB-H > CTX+NB-M > CTX+DHA-EE > CTX+NB-L.

### 2.4. Ovarian Hormone Levels in Serum and mRNA Expression Levels in Ovarian Tissue

**Figure 4:**
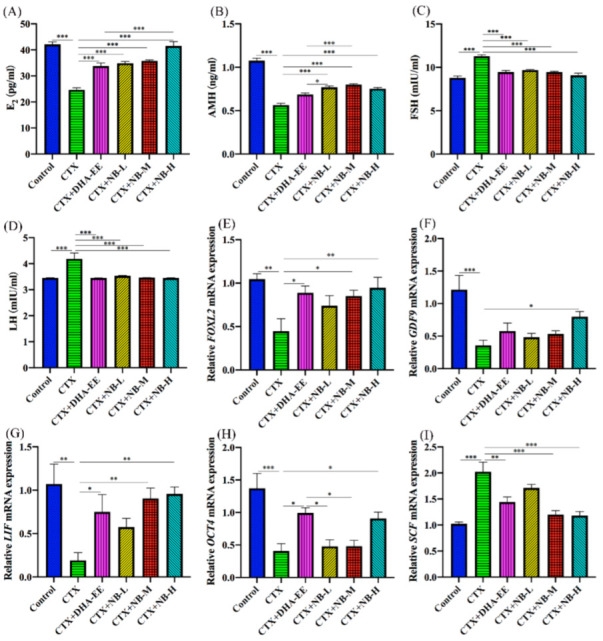
Effect of model and treatment on sex hormone levels and mRNA expression in mice. Sex hormone levels of E2 (**A**), AMH (**B**), FSH (**C**), and LH (**D**). mRNA expression levels of FOXL2 (**E**), GDF9 (**F**), LIF (**G**), OCT4 (**H**), and SCF (**I**) in mouse ovarian tissues, as determined by real-time PCR. * *p* < 0.05, ** *p* < 0.01, *** *p* < 0.005.

As shown in Figure 4A–D, serum gonadotropin levels, including E2 and AMH, were significantly lower in the CTX group than in the control group, whereas those of serum FSH and LH were significantly higher. Furthermore, the E2 level was significantly higher in all treatment groups compared with the CTX group. FSH and LH levels in all treatment groups and E2 levels in the CTX+NB-H group returned to normal. Additionally, the mRNA expression of five ovarian development-related genes (FOXL2, GDF9, LIF, OCT4, and SCF) were measured; FOXL2, OCT4, GDF9, and LIF were significantly downregulated (Figure 4E–H), whereas that of SCF was significantly upregulated in the CTX group compared with the control group (Figure 4I). Compared with those in the CTX group, the mRNA expression levels of FOXL2 and LIF in CTX+NB-M, FOXL2, GDF9, LIF, and OCT4 in CTX+NB-H, and FOXL2, LIF, and OCT4 in CTX+DHA-EE were significantly upregulated. SCF in CTX+NB-M, CTX+NB-H, and CTX+DHA-EE was significantly downregulated.

### 2.5. GC Apoptosis

**Figure 5:**
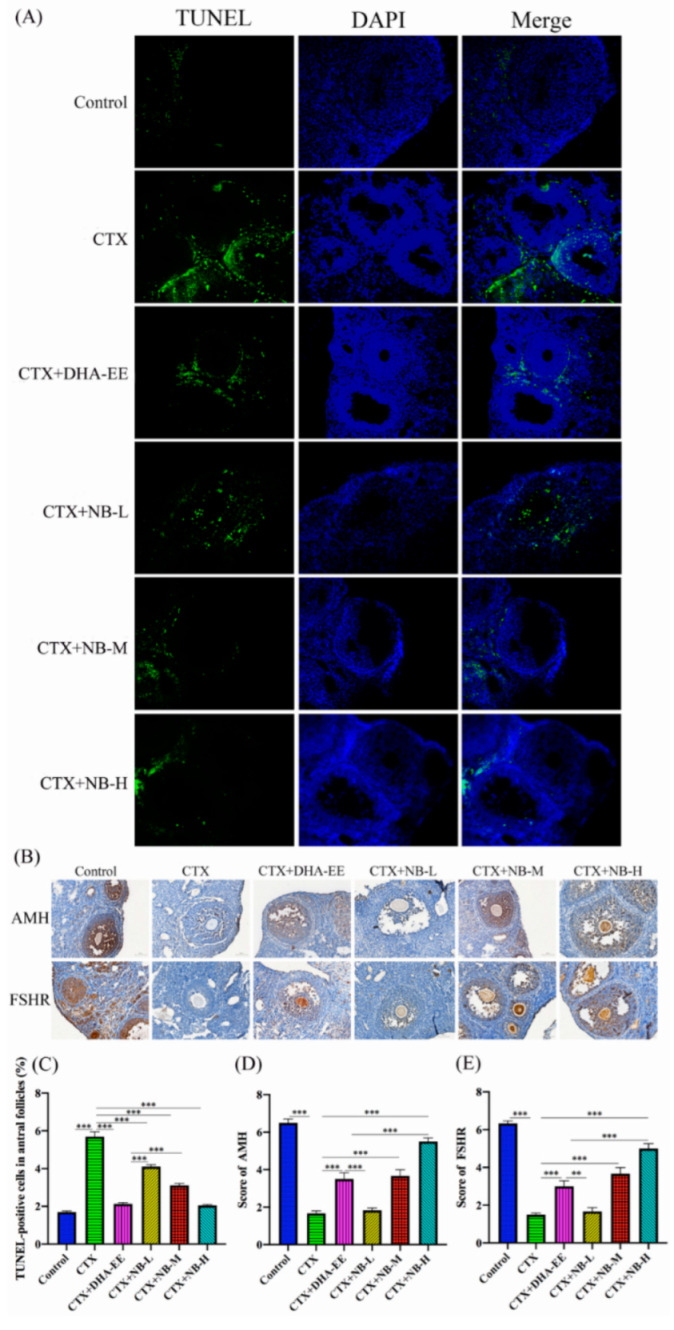
TUNEL and immunohistochemical analysis. Apoptosis of granulosa cells in ovarian tissues was measured via TUNEL assay (**A**). Green fluorescence: apoptotic cells; blue fluorescence: nucleus; magnification: 400×. The expression of AMH and FSHR in ovarian tissues were measured by immunohistochemical analysis (**B**). Blue: nucleus; brown: cells expressing AMH and FSHR in the cytoplasm; scale bar: 50 μm; magnification: 400×. TUNEL-positive cell ratios in the treatment groups were analyzed according to the number of TUNEL-positive granular cells among all granulosa cells in the follicle (**C**). Scoring of staining results (**D**,**E**). ** *p* < 0.01, *** *p* < 0.005.

To analyze ovarian cell apoptosis, the fractured DNA of apoptotic GCs in the antral follicles was observed using an in situ TUNEL assay. The number of apoptotic cells in the CTX group was significantly higher than that in the control group. However, after treatment with DHA-EE and NB-DHA, apoptosis of GCs was significantly decreased (Figure 5A). These results showed that apoptosis of GCs plays a crucial role in ovarian function development and growth in ovarian injured mice, and that DHA-EE and NB-DHA can restore ovarian function by inhibiting apoptosis. The proportion of TUNEL-positive cells decreased significantly after the additional administration of different doses of DHA-EE and NB-DHA (Figure 5C), especially in the CTX+NB-H and CTX+DHA-EE groups, in which the TUNEL-positive cell ratio was similar to that of the control group. This result reveals that high-dose NB-DHA can be considered suitable for the alleviation of CTX-induced ovarian cell apoptosis. DHA-EE also reduced GC apoptosis. The groups ranked as follows: CTX+NB-H > CTX+NB-M > CTX+DHA-EE > CTX+NB-L.

### 2.6. AMH and FSHR Expression in Ovaries

FSHR and AMH expression was detected by immunohistochemical analysis. Positive immunohistochemical results were scored. As shown in Figure 5B, the expression of AMH and FSHR in the CTX group was significantly lower than that in the control group. The expression of AMH and FSHR significantly increased after DHA-EE and NB-DHA treatment (Figure 5D,E). Notably, the effect of NB-H was better than that of DHA-EE at the same dosage. The treatments increased the expression of AMH and FSHR in the following order: CTX+NB-H > CTX+NB-M > CTX+DHA-EE > CTX+NB-L.

## 3. Discussion

Macamide-rich extract has been reported to stimulate the reproductive system, increase the number of mature follicular cells in female mice, and increase the number of sperm produced by male mice [25]. NB-DHA is a DHA derivative that belongs to the macamide family. It is difficult for higher plants to synthesize long-chain PUFAs, causing the content of NB-DHA in maca to be extremely low [14]. Therefore, in this study, DHA-rich fish oil was used as the starting material, and NB-DHA was efficiently synthesized with the carbodiimide condensation method [26]. Commercially available DHA is mainly in the form of ethyl esters or triglycerides. Theoretically, the degradation of the ester and benzylamide groups is different in vivo; as DHA-EE and NB-DHA are metabolized and absorbed at different rates and via different mechanisms, they might display different bioactive profiles in the human body [27,28,29]. 

After modeling with CTX, the mice lost their appetite and caused weight loss. After the experiment, the ovaries atrophied and the weight of the ovaries decreased, causing ovarian injury. Compared with the CTX group, the body and ovarian weights of the mice increased significantly after the administration of CTX+NB-M and CTX+NB-H, and CTX+DHA-EE treatment has just stabilized their body weight. We speculated that CTX caused changes in the ovarian microenvironment in mice, causing ovarian damage and leading to ovarian atrophy, while DHA-EE and NB-DHA alleviated CTX-induced ovarian injury. Our results indicate that both DHA-EE and NB-DHA reverse ovarian injury by increasing the number of normal follicles and decreasing that of atretic follicles. Ovarian injury clinically manifests as an abnormal estrous cycle and ovarian function, and severe injury may lead to premature ovarian failure and infertility [30,31,32]. CTX-induced ovarian injury mice in the CTX group exhibited an irregular estrous cycle and changes in ovarian function indicators. The primordial follicles, which act as the initial unit of follicle maturation or generation, undergo a series of developmental stages that form primary, secondary, and mature follicles, which then release the oocytes for reproduction. Most primitive follicles eventually become closed follicles, and only a few reach maturity [33,34]. Therefore, the growth of primordial follicles is related to the development of the entire ovary. Ovarian injury can cause a decrease in AMH and E2 levels and an increase in FSH and LH levels, which are considered four important indices for the evaluation of ovarian injury and abnormal follicular maturation. Our results indicated that the irregular estrous cycles of mice in the CTX+DHA-EE, CTX+NB-M, and CTX+NB-H groups were positively regulated to close the regular cycle of mice in the control group with a prolonged estrus period and shortened late estrus and interval. CTX-induced ovarian injury resulted in enhanced serum FSH and LH levels and decreased serum E2 and AMH levels. Meanwhile, the number of primordial, primary, and secondary follicles was reduced, and the number of atresia follicles increased. However, the administration of DHA-EE and different doses of NB-DHA to alleviate this injury increased the number of primordial, primary, and secondary follicles, reduced the number of atretic follicles, increased serum AMH and E2 levels, and reduced FSH and LH levels. These results reveal the regulatory function of both DHA-EE and NB-DHA on the estrous cycle of mice with ovarian injury caused by CTX.

The formation, maturation, and growth of follicles are regulated by many intra- and extra-ovarian factors. FOXL2 is involved in multiple dysfunctional states in the ovary and is essential for GC differentiation and maintenance of ovarian function, and is expressed in GCs with low differentiation in small- and medium-sized follicles [35]. GDF9 encodes a protein secreted into the follicle by oocytes [36] and plays a pivotal role in optimizing the oocyte microenvironment and growth, development, atresia, ovulation, fertilization, and normal reproduction of the follicle [37]. It also has a role in promoting the proliferation and apoptosis of GCs while stimulating the expression of Kit ligands on GCs. LIF is expressed in the ovaries and promotes follicle growth. LIF has also been shown to coordinate follicular growth and ovulation sequences and can locally regulate follicular growth [38]. Oct4 has the potential to recruit mature oocytes. Overexpression in ovarian stem/stromal cells enhances oocyte-like differentiation in vitro and follicle formation in vivo [39]. SCF is essential for the early follicular development. It stimulates stromal cell function and promotes follicular growth through the Erk1/2 pathway, and can be used as a crucial regulator of embryo and ovarian growth to exert its biological effects [40,41]. CTX can cause ovarian injury and disorders in the expression levels of these mRNA [40]. Our results also revealed that the mRNA levels of FOXL2, GDF9, LIF, and OCT4 were decreased, while those of SCF were increased in the CTX group. After high-dose NB-DHA treatment, the levels of FOXL2, GDF9, LIF, and OCT4 increased, whereas those of SCF decreased. The mechanism of action of NB-DHA might be to promote the growth and maturation of follicles via the regulation of the mRNA expression levels of these five genes, thereby reversing ovarian injury caused by CTX and protecting the ovaries. At the same dose, the effect of NB-H was better than that of DHA-EE. 

According to previous reports, follicular atresia occurs when more than 10% of GCs undergo apoptosis. Follicular atresia can cause a decline in ovarian function [42,43]. Our results indicated that apoptotic GCs and atretic follicles were significantly increased in the CTX group and reduced after both DHA-EE and NB-DHA treatment, whereas that of other types of follicles increased, suggesting that NB-DHA acts on AMH expression through cytokines secreted by granulocytes. AMH is produced by GCs of early ovarian developing follicles and is expressed at high levels throughout follicle formation. When ovarian GCs undergo apoptosis, DNA is fragmented and 3′-OH is combined with TdT to generate fluorescence. Our results analyzed the ratio of fluorescent cells and showed that CTX induced apoptosis of ovarian cells, which was reversed by DHA-EE and NB-DHA administration. In this case, we infer that DHA-EE and NB-DHA can reduce the apoptosis of ovarian granulosa cells, thereby achieving the effect of protecting the ovary. However, the anti-apoptotic activity of NB-DHA remains to be explored. The serum AMH level may represent the quantity and quality of the follicular pool, which is related to ovarian aging and failure, and reflects the state of the ovaries. Follicles are surrounded by GCs instead of membranous cells, oocytes, and ovarian stromal cells [44,45]. In addition, there is no expression of AMH when the follicle is atresia [43]. FSHR is expressed specifically in the GCs of the ovary and plays a key role in follicular function by interacting with its ligand FSH in the ovaries. When the follicle is atresia, FSHR expression is downregulated [46]. The immunohistochemical analysis results showed that both AMH and FSHR were expressed in secondary follicles, and the serum levels of AMH and FSHR were consistent with those in the ovary. The expression of AMH and FSHR in the CTX group was significantly lower than that in the other groups. The levels of both recovered after NB-DHA treatment, indicating that NB-DHA facilitates the growth of GCs and the expression of AMH and FSHR, thereby promoting follicle growth. The decreased primary follicles in the mouse model of ovarian injury, potentially owing to decreased serum AMH levels, lead to premature depletion of the original follicular pool. After treatment with NB-DHA, the levels of AMH and FSHR increased, indicating that NB-DHA increased the number of primordial follicles, reduced the failure of the primordial follicle pool caused by CTX, restored GC growth, and restored ovarian function. However, DHA-EE at the same dose as high-dose NB-DHA had no significant effect on AMH and FSHR expression in the follicles. 

## 4. Materials and Methods

### 4.1. Materials

Fish oil (DHA content > 80%) was obtained from Shanxi Taike Biotech Co., Ltd. (20200712-002, Xi’an, China). Rhizomucor miehei lipase (L8621) was obtained from Solarbio (Beijing, China). Ethyl dimethylaminopropyl carbodiimide (EDC), benzylamine, dichloromethane, HOBt ·H_2_O, and triethylamine were obtained from Aladdin Co., Ltd. (Shanghai, China). The 3,3-diaminobenzidine kit (20×) (CW0125) was obtained from Cwbio Co., Ltd. (Beijing, China). Proteinase K (BL104A) and anti-fluorescence quenching mounting fluid (BL701A) were obtained from Biosharp Co., Ltd. (Hefei, China). Anti-FSHR (#40941) and anti-Müllerian-inhibiting factor (#42063) polyclonal antibodies were obtained from SAB Co., Ltd. (Baltimore, MD, USA).

Enzyme-linked immunosorbent assay (ELISA) kits for luteinizing hormone (LH; CSB-E12770m), follicle-stimulating hormone (FSH; CSB-E06871m), estradiol (E2; CSB-E05109m), and anti-Müllerian hormone (AMH; CSB-E13156m) were obtained from Cusabio Biotech Co., Ltd. (Wuhan, China). Evo M-MLV RT Mix Kit with gDNA Clean for qPCR (AG11728) and SYBR Green Pro Taq HS premixed qPCR kits (AG11701) were obtained from AgBio Co., Ltd. (Changsha, China). A TUNEL kit (in situ cell death detection; C1086) and DAPI (4′,6-diamidino-2-phenylindole; C1005) were obtained from Beyotime Biotech Co., Ltd. (Shanghai, China).

### 4.2. Synthesis and Purification of DHA-EE and NB-DHA

Twenty milliliters of fish oil were added to 20 mL of 10% (*w*/*v*) lipase solution, mixed homogeneously, and hydrolyzed at 45 °C for 24 h. The oil layer was collected, washed alternately with distilled water (40 mL) and n-hexane (40 mL), and the aqueous layer was discarded. The supernatant was concentrated using a rotating vacuum evaporator at 45 °C for 30 min. The fatty-acid-rich residues were stored frozen for subsequent synthesis experiments. DHA-EE was synthesized using a transesterification method. In brief, free fatty acids (700 μL) were mixed with 500 μL of NaOH-ethanol solution at 70 °C for 30 min, washed twice with saturated NaCl solution, and centrifuged at 5000× *g* for 10 min to collect the oil layer containing DHA-EE. NB-DHA was synthesized using the carbodiimide condensation method. Briefly, 100 mL of dichloromethane, 700 μL of free fatty acids, 528.6 μL of triethylamine, 0.206 g of HOBt H_2_O, and 0.292 g of EDC were mixed and agitated at 25 °C for 20 h, and 166 μL of benzylamine was added and stirred at 25 °C for 4 h. Subsequently, 200 mL of 10% HCl was added to the residue after drying, and 200 mL n-hexane was added, mixed homogeneously, and rested for 10 min. The upper layer was collected and washed alternately with 10% HCl and 10% NaOH to remove macroscopic impurities. Finally, DHA-EE and NB-DHA were purified according to our previous method, and their purities were analyzed by HPLC [14].

### 4.3. Animals and Treatment

Healthy female mice (20 ± 2 g, 7–8 weeks old, C57BL/6) were obtained from the Guangdong Medical Laboratory Animal Center (Guangzhou, China). The mice were kept under pathogen-free conditions in a temperature (23 ± 2 °C) and humidity (55% ± 15%) control system, and all animal facilities were kept in a 12 h light–dark cycle. Food and water were provided free access for one week prior to the experiment. A preliminary experiment was carried out to determine the effective dose range of DHA-EE and NB-DHA. The mice were randomly divided into six independent groups (n = 6): control, ovarian injury model caused by CTX, CTX+DHA-EE (100 mg/kg/day), CTX+low-dose NB-DHA (CTX+NB-L, 25 mg/kg/day), CTX+medium-dose NB-DHA (CTX+NB-M, 50 mg/kg/day), and CTX+high-dose NB-DHA (CTX+NB-H, 100 mg/kg/day) groups. All drugs were administered to the mice after dissolving in Tween 80 solution at a concentration of 1%. Mice in the DHA-EE and NB-DHA groups were gavaged once a day from day 1 to day 21. The CTX, CTX+NB-DHA, and CTX+DHA-EE groups were injected intraperitoneally with CTX (200 mg/kg) on the eighth day after adaptive feeding. The control group was fed normally without any drugs until the end of the experiment. All treatments were started at the same time and were sustained for 21 days. The survival rate of the mice was 100% during the experiments.

### 4.4. Ovarian Index and Estrous Cycle Examination

The mice were weighed prior to euthanasia. The isolated ovaries were repeatedly rinsed with precooled sterile saline, blotted dry with filter paper, and weighed. Nucleated cells, keratinized epithelial cells, and leukocytes in vaginal smears were observed under a light microscope, and the stages of the estrous cycle, including proestrus, estrus, metestrus, and diestrus phases, were determined based on the identification and proportions of cells. The estrous cycle was monitored continuously for 21 d.

### 4.5. Morphological Analysis and Follicle Counting

The ovaries were fixed with paraformaldehyde solution (4%) for 12 h and then washed with running water for 12 h. Subsequently, ovaries were dehydrated, embedded in paraffin, and stored at −20 °C. The tissues were sliced serially (4 μm thick), and one every five sheets was selected for hematoxylin and eosin (H&E) staining. Filming was performed using a slide scanning system (SQS-40P, Teksqray, Shenzhen, China). The viewing angle was determined under a microscope at low magnification, while primordial, primary, secondary, and atretic follicles were counted at high magnification. Six ovarian samples were randomly selected from each group, and sections were observed in 3 views under 400× to count follicles at all stages.

### 4.6. ELISA

The mice were fasted for 8 h after the final administration. Blood samples were collected from the eye veins, placed in anticoagulation tubes, and centrifuged at 4000× *g* for 15 min. The levels of serum FSH, LH, E2, and AMH were measured using an ELISA kit.

### 4.7. RNA Extraction and Reverse-Transcription qPCR

Total RNA was extracted from ovarian tissues using an RNA extraction kit. The RNA concentration was 500–1000 ng/μL. Then, 1 μg of RNA was reverse transcribed into cDNA, as required by the reverse transcription kit. The qPCR kit was used to measure the expression levels of GAPDH, FOXL2, GDF9, LIF, OCT4, and SCF. The primer sequences were as follows:

GAPDH-forward primer: 5′-TGTGTCCGTCGTGGATCTGA-3′, 

GAPDH-reverse primer: 5′-TTGCTGTTGAAGTCGCAGGAG-3′; 

FOXL2-forward primer: 5′-CACCTCCAGGCCAGGTCTTTA-3′,

FOXL2-reverse primer: 5′-TTTAGCAAACTCCAAGGCCATTAC-3′;

GDF9-forward primer: 5′-GTTCCCAAACCCAGCAGAAGTC-3′,

GDF9-reverse primer: 5′-GTCCAGGTTAAACAGCAGGTCCA-3′;

LIF-forward primer: 5′-TTGATCCCGACTCAAGCAACC-3′, 

LIF-reverse primer: 5′-CTGAAGCCGCTACCATGCAA-3′; 

OCT4-forward primer: 5′-CAGACCACCATCTGTCGCTTC-3′,

OCT4-reverse primer: 5′-AGACTCCACCTCACACGGTTCTC-3′;

SCF-forward primer: 5′-AGATCTGCGGGAATCCTGTGA-3′, 

SCF-reverse primer: 5′-CATCCCGGCGACATAGTTGA-3′.

### 4.8. In Situ Cell Death Detection

For the in situ TUNEL paraffin staining, a part of each ovarian sample slice was randomly selected (n = 6). The sections were covered with protease K solution (20 mg/mL) and incubated in a wet chamber at 37 °C for 30 min. The sections were washed five times with PBS and then covered with Triton X-100 (1%) at 4 °C for 10 min. An in situ cell death assay kit was used for the TUNEL assay. The sections were incubated in TUNEL reaction mixture (TdT enzyme and fluorescent-labeled buffer) for 60 min at 37 °C under dark and humid conditions to capture the fragmented DNA of apoptotic cells. The sections were then incubated with DAPI at 24 °C for 5 min, washed with PBS, dried around the tissues, mounted with an anti-fluorescence quencher, and observed under a fluorescence microscope (BX53, Olympus, Tokyo, Japan). Whether the section was intact was established at 100× magnification, and the apoptosis of atretic follicles was carefully observed at 400× magnification. ImageJ 1.53a software was used to analyze the proportion of TUNEL-positive cells in the antral follicles.

### 4.9. Immunohistochemistry

Paraffin-embedded tissue sections were dewaxed in a microwave oven for antigen repair. Immunohistochemical staining was performed using an SP immunohistochemistry kit. Rabbit anti-AMH (1:100) and anti-FSHR (1:150) antibodies were incubated with the tissue at 4 °C for 12 h. Six areas on each slide were randomly selected for inspection and filmed using the SQS-40P slide scanning system. The German immune response scoring standard (IRS) was used to score the staining results [47]. 

### 4.10. Data Analysis

All data were analyzed using GraphPad Prism 8 software, and the results are shown as the mean ± standard error of the mean (SEM). One-way analysis of variance was used to evaluate statistical significance among the experimental groups. All data were considered statistically significant at * *p* < 0.05, ** *p*< 0.01, and *** *p*< 0.005.

## 5. Conclusions

In summary, these data demonstrate that NB-DHA alleviates ovarian injury in mice. NB-DHA reverses the high levels of gonadotropins and low levels of estrogen in the serum of mice with ovarian injury, promotes follicular development, inhibits follicular atresia and GC apoptosis via the upregulation of AMH and FSHR expression in GCs, and regulates the mRNA expression levels of ovarian-related genes to increase the ovarian reserve capacity. Natural DHA can be used as a beneficial dietary supplement to improve ovarian function, and NB-DHA is a promising compound for the clinical treatment of patients with ovarian injury. 

## Data Availability

Not available.

## Sample Availability

Samples of the compounds *N*-Benzyl Docosahexaenamide are available from the authors.
